# Supplementary material for: From sequence to enzyme mechanism using multi-label machine learning
Source: BMC Bioinformatics. 2014 May 19;15:150. doi: 10.1186/1471-2105-15-150 (PMC4229970; doi:10.1186/1471-2105-15-150)
Supplement: Additional file 2 — Java code of ml2db. Additional file ml2db_code.tar.gz contains the Java source code to run the multi-label machine learning experiments and save the results to database. The code’s Javadoc is included. [file 1471-2105-15-150-S2.zip › additional file 2/ml2db/utils/doc/uk/ac/standrews/utils/main/database/class-use/DbManager.html]

Uses of Class uk.ac.standrews.utils.main.database.DbManager


JavaScript is disabled on your browser.


- Overview
- Package
- Class
- Use
- Tree
- Deprecated
- Index
- Help

- Prev
- Next

- Frames
- No Frames

- All Classes

## Uses of Class uk.ac.standrews.utils.main.database.DbManager

- Packages that use DbManager

  | Package | Description |
  | --- | --- |
  |  |  |
  | --- | --- |
  | uk.ac.standrews.utils.main.collectionutils.setutils |  |
  | uk.ac.standrews.utils.main.database |  |
  | uk.ac.standrews.utils.main.setutils |  |
  | uk.ac.standrews.utils.test.database |  |
- - ### Uses of DbManager in uk.ac.standrews.utils.main.collectionutils.setutils

    Constructors in uk.ac.standrews.utils.main.collectionutils.setutils with parameters of type DbManager

    Constructor and Description

    `SupersetsManager(java.lang.String sqlQuery,
    DbManager dbManager,
    java.lang.String separator)`
  - ### Uses of DbManager in uk.ac.standrews.utils.main.database

    Subclasses of DbManager in uk.ac.standrews.utils.main.database

    | Modifier and Type | Class and Description |
    | --- | --- |
    |  |  |
    | --- | --- |
    | `class` | `TableCreator` Class to create a table object in the database. |
    | `class` | `TableManager` Class to create/read/write to a database table. |
    | `class` | `TableReader` Class |
    | `class` | `TableRow` Contains the data for a generic row (a list of [table column, value] entries) to be written to a database table. |
    | `class` | `TableWriter` A TableWriter object contains all the information to create, fill and delete a database table. |

    Methods in uk.ac.standrews.utils.main.database that return DbManager

    | Modifier and Type | Method and Description |
    | --- | --- |
    |  |  |
    | --- | --- |
    | `DbManager` | Table.`getDbManager()` public int saveRowToTable(TableRow row) { // write row to table TableWriter writer = this.getTableWriter(); int resultId = writer.insertReturnsKey(row); if (m\_debug ) { System.out.println("Written row with id: " + resultId + " to table " + this.getTableName() + " database " + this.getDbManager().getDatabaseName() + " - " + this.getClass().getName()); } return resultId; } |
    | `DbManager` | DbManaged.`getDbManager()` |

    Methods in uk.ac.standrews.utils.main.database with parameters of type DbManager

    | Modifier and Type | Method and Description |
    | --- | --- |
    |  |  |
    | --- | --- |
    | `void` | Table.`initialise(java.lang.String tableName, DbManager dbManager)` |
    | `void` | Table.`initialise(java.lang.String tableName, DbManager dbManager, java.util.Vector<TableColumn> columns)` |

    Constructors in uk.ac.standrews.utils.main.database with parameters of type DbManager

    Constructor and Description

    `DbCreator(DbManager dbManager)`

    `DbManaged(DbManager dbManager)`

    `DbManager(DbManager dbManager)`

    Constructor 2: creates the database manager from another one

    `DbReader(DbManager dbManager)`

    `DbWriter(DbManager dbManager)`

    `Table(java.lang.String tableName,
    DbManager dbManager)`

    Construct a table read-only.

    `Table(java.lang.String tableName,
    java.util.Vector<TableColumn> columns,
    DbManager dbManager)`

    Construct a table for writing to: the columns definitions (name, data type) must be known.
  - ### Uses of DbManager in uk.ac.standrews.utils.main.setutils

    Constructors in uk.ac.standrews.utils.main.setutils with parameters of type DbManager

    Constructor and Description

    `SupersetsManager(java.lang.String sqlQuery,
    DbManager dbManager,
    java.lang.String separator)`
  - ### Uses of DbManager in uk.ac.standrews.utils.test.database

    Methods in uk.ac.standrews.utils.test.database that return DbManager

    | Modifier and Type | Method and Description |
    | --- | --- |
    |  |  |
    | --- | --- |
    | `static DbManager` | DbManagerTest.`getDataDbManager()` |
    | `static DbManager` | DbManagerTest.`getTestDbManager()` |

- Overview
- Package
- Class
- Use
- Tree
- Deprecated
- Index
- Help

- Prev
- Next

- Frames
- No Frames

- All Classes
